# Anlotinib plus camrelizumab achieved long‐term survival in a patient with metastatic esophageal neuroendocrine carcinoma

**DOI:** 10.1002/cnr2.1855

**Published:** 2023-06-28

**Authors:** Lingxiao Zhou, Guanxin Xu, Tianwei Chen, Qiyuan Wang, Jing Zhao, Ting Zhang, Rong Duan, Yang Xia

**Affiliations:** ^1^ Key Laboratory of Respiratory Disease of Zhejiang Province, Department of Respiratory and Critical Care Medicine Second Affiliated Hospital of Zhejiang University School of Medicine Hangzhou Zhejiang China; ^2^ Cancer Center Zhejiang University Hangzhou Zhejiang China; ^3^ Department of Thoracic Surgery Second Affiliated Hospital of Zhejiang University School of Medicine Hangzhou Zhejiang China; ^4^ Department of Radiology Second Affiliated Hospital of Zhejiang University School of Medicine Hangzhou Zhejiang China; ^5^ Department of Medical Oncology Second Affiliated Hospital of Zhejiang University School of Medicine Hangzhou Zhejiang China; ^6^ Department of Radiation Oncology Second Affiliated Hospital of Zhejiang University School of Medicine Hangzhou Zhejiang China; ^7^ Department of Pathology Second Affiliated Hospital of Zhejiang University School of Medicine Hangzhou Zhejiang China

**Keywords:** antiangiogenic therapy, combination therapy, esophageal neuroendocrine carcinoma, immune checkpoint inhibitor, metastasis

## Abstract

**Background:**

Esophageal neuroendocrine carcinoma (NEC) is a rare cancer with an extremely poor prognosis. The average overall survival of patients with metastatic disease is only 1 year. The efficacy of anti‐angiogenic agents combined with immune checkpoint inhibitors remains unknown.

**Case Presentation:**

A 64‐year‐old man, initially diagnosed with esophageal NEC, underwent neoadjuvant chemotherapy and esophagectomy. Although the patient remained disease‐free for 11 months, eventually the tumor progressed and did not respond to three lines of combined therapy (etoposide plus carboplatin with local radiotherapy, albumin‐bound paclitaxel plus durvalumab, and irinotecan plus nedaplatin). The patient then received anlotinib plus camrelizumab, and a dramatic regression was observed (confirmed by positron emission tomography‐computed tomography). The patient has been disease‐free for over 29 months and has survived for over 4 years since diagnosis.

**Conclusion:**

Combined therapy with anti‐angiogenic agents and immune checkpoint inhibitors may be a promising strategy for esophageal NEC, although more evidence is warranted to validate its efficacy.

## 1 INTRODUCTION

Esophageal neuroendocrine carcinoma (NEC) is much rarer than typical esophageal carcinomas with a worse prognosis, and a median overall survival (OS) of 12–13 months in stage IV patients.^1^, ^2^, ^3^ There are no standard therapeutic strategies for treating esophageal NEC.^4^ For patients with stage IV or relapsed NEC, systemic and local treatment must be considered. Chemotherapy is the first choice of treatment, which is similar to small cell lung carcinoma (SCLC).^3^

In recent years, anti‐angiogenic agents, have exhibited potential for SCLC management.^5^ Programmed cell death protein 1 (PD‐1) and programmed cell death protein ligand 1 (PD‐L1) inhibitors have also been widely studied for the treatment of extensive‐stage SCLC (ES‐SCLC).^6^, ^7^ Here, we report our experience with a patient with esophageal NEC, in whom several lines of chemotherapy‐based treatment failed but complete disease regression was observed after treatment with anti‐angiogenic therapy and PD‐1 inhibitors, which may be a promising treatment strategy for this unique cancer.

## 2 CASE DESCRIPTION

A 64‐year‐old Chinese man with no notable family history was admitted to the Second Affiliated Hospital of Zhejiang University School of Medicine on November 16, 2018. The patient had difficulty in swallowing, but no chest pain, nausea or vomiting. Gastroscopic biopsy indicated esophageal NEC. Positron emission tomography‐computed tomography (PET‐CT) revealed slight fluorodeoxyglucose uptake in the mid‐esophagus and lymph nodes around the superior mediastinum. The patient underwent esophagectomy on January 3, 2019 after two cycles of neoadjuvant chemotherapy with etoposide (100 mg/m^2^) and carboplatin [AUC × (GFR + 25), AUC = 5]. Pathological examination of the resected specimen confirmed the diagnosis of EGFR‐positive esophageal NEC (NET G3) with lymph node metastasis (T2N1M0, stage IIB).

**FIGURE 1:**
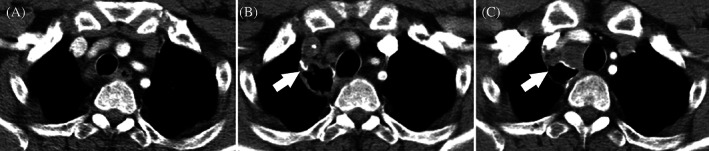
Chest CT images before metastatic esophageal NEC was confirmed. (A) Cancer first diagnosed on November 16, 2018. (B) Assessment after neoadjuvant chemotherapy and esophagectomy on 27 February 2019, and no enlarged lymph node detected. (C) Enlarged 2R lymph node in December 8, 2019.

**FIGURE 2:**
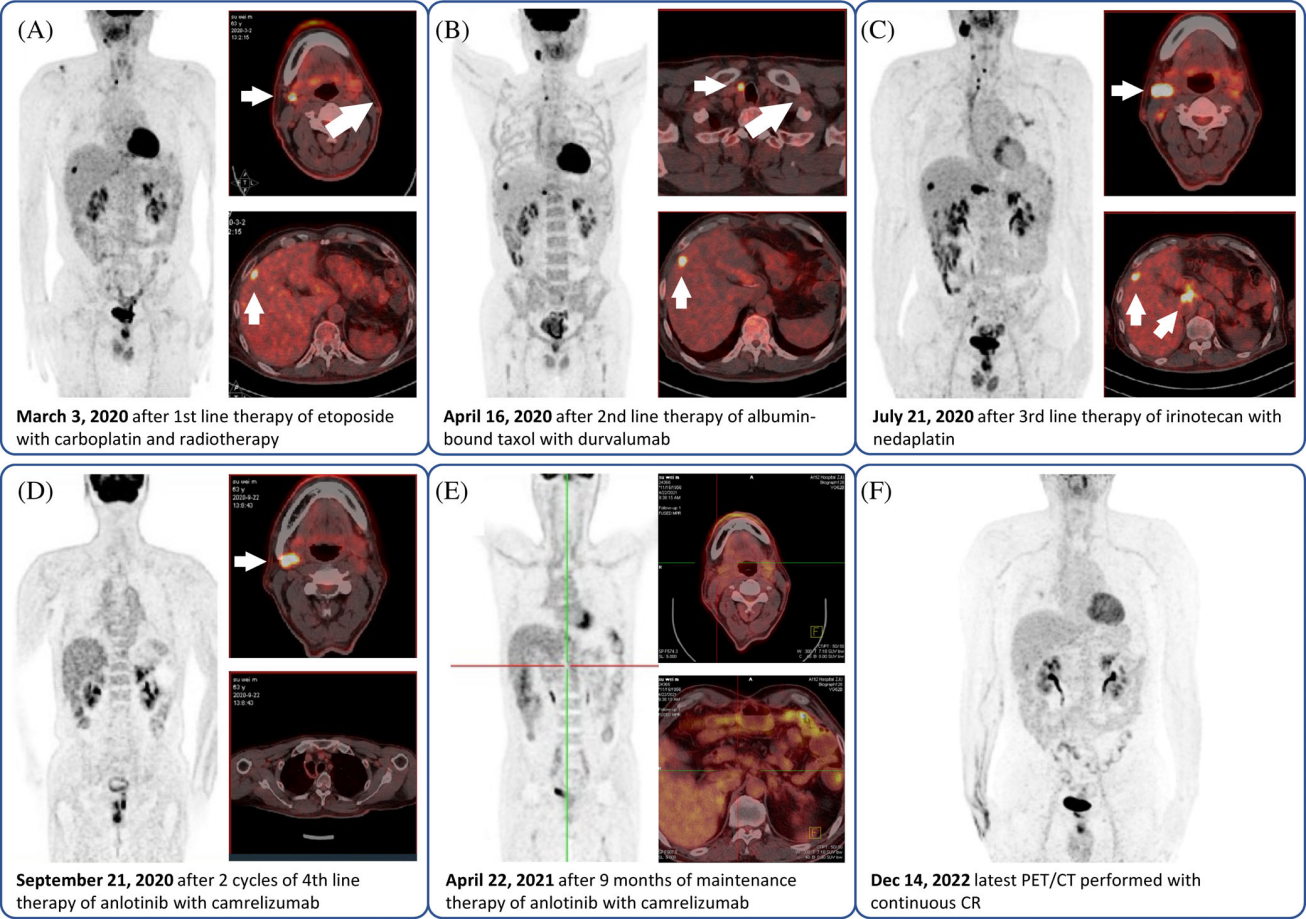
PET/CT images and treatment throughout the disease course after metastasis revealed in December 2019. (A) Metastasis in cervical lymph node and liver. (B) Metastasis in mediastinal lymph node and liver. (C) Metastasis in cervical lymph node, liver and retroperitoneal lymph node. (D) Metastasis detected only in cervical lymph node. (E, F) No metastasis detected.

The patient remained disease‐free for 11 months. On December 8, 2019, a chest CT indicated an enlarged 2R lymph node (Figure 1). Chemotherapy with etoposide (100 mg/m^2^) and carboplatin (AUC = 5) and radiotherapy (mediastinal lymph nodes, D95 pGTV 57.5 Gy/25 F; supraclavicular and subclavicular lymph nodes, D95 PTV 50 Gy/25 F) were administered from December 2019 to February 2020. Liver metastasis and cervical lymph node metastasis were found on March 3, 2020 on PET‐CT. Two cycles of second‐line albumin‐bound paclitaxel (chemotherapy, 260 mg/m^2^) plus durvalumab (a PD‐L1 inhibitor, 10 mg/m^2^) were administered in March 2020. However, PET‐CT indicated that the metastatic liver lesion had increased in size. The patient underwent third‐line chemotherapy with irinotecan (65 mg/m^2^) and nedaplatin (80 mg/m^2^) from April 2020 to June 2020. However, a new metastatic cervical region II lymph node and an enlarged retroperitoneal lymph node were noted on June 16, 2020. Furthermore, needle biopsy of the cervical region II lymph nodes confirmed metastatic carcinoma. Fourth‐line anlotinib (an anti‐angiogenic agent, 12 mg daily for 2 weeks in a 3‐week cycle) and camrelizumab (a PD‐1 inhibitor, 200 mg every 3 weeks) were initiated in July 2020. This combination regimen had significant efficacy, with an impressive decrease in the fluorodeoxyglucose uptake in the cervical region II lymph node and mediastinal 2R lymph node 2 months later. These metastases showed regression on follow‐up PET‐CT. Anlotinib and camrelizumab were administered as maintenance therapies. To date, the patient's progression‐free survival (PFS) is >29 months, and his overall survival (OS) is >50 months, with continuous complete regression confirmed by PET‐CT on December 14, 2022 (Figure 2).

## 3 DISCUSSION

To the authors' knowledge, the survival period of this patient is the longest among the reported cases. Inspired from the development of SCLC treatment, the combination treatment of anti‐angiogenic agents and immunotherapy was chosen for the patient and surprisingly showed excellent therapeutic effect in this single case, though it is not a recommended strategy for advanced esophageal NEC. Currently, the treatment strategy for esophageal NEC is based on the reported cases and clinical experience of other NECs, especially SCLC. Systemic chemotherapy combined with local treatment may be the optimal treatment for unresectable esophageal NEC, and platinum‐based two‐drug chemotherapy is generally recommended.^3^, ^8^, ^9^ In recently reported cases of unresectable esophageal NEC, patients still received chemoradiotherapy plus local treatment as the only treatment,^10^, ^11^, ^12^ which does not improve survival.

Not only esophageal NEC, similar difficulties are encountered in the management of SCLC, before several new agents proven efficacious in the last 5 years. Two PD‐L1 inhibitors, durvalumab and atezolizumab, improved OS for patients with ES‐SCLC patients (CASPIAN and IMPOWER 133).^6^, ^7^ As an anti‐angiogenic agent, anlotinib is another therapeutic alternative for third‐line SCLC treatment.^5^ The combination of anti‐angiogenic agents and immunotherapy for SCLC treatment has been attempted. A phase 2 trial conducted by Fan et al.^13^ examining camrelizumab plus apatinib in 59 patients with ES‐SCLC who failed chemotherapy reached a median PFS of 3.6 months. Preclinical studies have attempted to elucidate the possible mechanisms of dual therapy. Zhao et al.^14^ found that apatinib could modulate the immunosuppressive tumor microenvironment to reduce resistance to anti‐PD‐1/PD‐L1 treatment. Zhang et al.^15^ discovered that dual therapy could promote antitumor immunity in gastric cancer. PD‐L1 could also be significantly upregulated in anlotinib‐treated renal cell carcinoma cells.^16^ Since NECs in the esophagus and lungs share similar pathologic and clinical manifestations, it is reasonable to propose that anti‐angiogenic agents and immunotherapy might also benefit patients with esophageal NEC. In the case of resistance of multiple chemotherapy agents, we provided anti‐angiogenic agents and immunotherapy for this patient. Our patient has already reached a remarkable PFS of over 29 months with anlotinib and camrelizumab combination therapy, which is much longer than the chemotherapy‐based systemic treatment in other reported cases. However, there have been very few attempts at this combination treatment and the efficacy still requires more cases to confirm.

## 4 CONCLUSION

We encountered a case of metastatic esophageal NEC in which complete regression and long survival were observed after treatment with combination of anlotinib and camrelizumab, which can be hardly achieved in routine treatment. Given the poor prognosis of the disease, this therapy should be considered for patients with unresectable esophageal NEC, especially for who who have developed chemoresistance.

## AUTHOR CONTRIBUTIONS

All authors had full access to the data in the study and take responsibility for the integrity of the data and the accuracy of the data analysis. Conceptualization, Lingxiao Zhou and Yang Xia. Project Administration, Yang Xia, Guanxin Xu, Qiyuan Wang, Jing Zhao, Ting Zhang, and Rong Duan. Data Curation, Lingxiao Zhou and Tianwei Chen. Writing – Original Draft Preparation, Lingxiao Zhou. Writing – Review & Editing, Yang Xia. All authors contributed to the article and approved the submitted version.

## CONFLICT OF INTEREST STATEMENT

The authors declare that the research was conducted in the absence of any commercial or financial relationships that could be construed as a potential conflict of interest.

## ETHICS STATEMENT

Approval of the research protocol by an Institutional Reviewer Board: This study was reviewed and approved by Ethics Committee of the Second Affiliated Hospital of Zhejiang University School of Medicine and it conforms to the provisions of the Declaration of Helsinki. Written informed consent was obtained from the patient for publication of this case report and any accompanying images.

## Data Availability

Data will be made available on request.
